# Survival Mediterranean Style: Lifestyle Changes to Improve the Health of the US Fire Service

**DOI:** 10.3389/fpubh.2017.00331

**Published:** 2017-12-18

**Authors:** Maria Korre, Mercedes Sotos-Prieto, Stefanos N. Kales

**Affiliations:** ^1^Environmental & Occupational Medicine & Epidemiology Program, Department of Environmental Health, Harvard T.H. Chan School of Public Health, Boston, MA, United States; ^2^Cambridge Health Alliance, Harvard Medical School, Cambridge, MA, United States; ^3^The Diabetes Institute, Ohio University, Athens, OH, United States; ^4^Department of Food Sciences and Nutrition, School of Applied Heath Sciences and Wellness, Ohio University, Athens, OH, United States

**Keywords:** Mediterranean diet, lifestyle, firefighters, workplace health, obesity, cardiovascular health, cancer

## Abstract

Cardiovascular disease (CVD) causes almost half of all on-duty deaths in US firefighters and is an important and costly cause of morbidity. In addition, cancer is a growing health concern in this population. Obesity and obesity-associated, cardiometabolic risk clustering are major, modifiable risk factors for fire service CVD and cancer risk. The Mediterranean diet (MedDiet) is proven effective in primary and secondary CVD prevention. It is also associated with a decreased risk of cancer and other chronic diseases. Moreover, it can be adapted into successful workplace interventions. Emerging data from our group regarding the US Fire Service show that greater compliance with the MedDiet is associated with improved CVD risk profiles and less weight gain among career firefighters. Moreover, the fact that career firefighters take a considerable number of meals communally on the job also represents an excellent opportunity for a workplace Mediterranean Diet Nutritional Intervention (MDNI). The devastating effects of obesity, CVD, and cancer on the US fire service are recognized, but currently few effective preventive programs exist. The consistently positive health benefits from following a MedDiet and promising preliminary data in the fire service justify translational research to determine the most effective means of delivering MDNIs to US firefighters. Therefore, a high priority should be assigned to efforts, which can help further disseminate and implement our program of novel behavior change strategies, “Survival Mediterranean Style,” throughout the US fire service and eventually to other occupations.

## Introduction

Today, as a result of the worldwide epidemics of obesity and diabetes, we are witnessing a strong and renewed interest in the traditional Mediterranean diet (MedDiet). A nutritional approach that derives its appeal not only from its many proven health benefits but also from its delicious meals combining diverse flavors, colors, and aromas fresh from the land and sea.

The MedDiet has consistently been associated with decreased all-cause mortality, less chronic disease, and better quality of life (1–6). Robust evidence in the general population consistently demonstrates the benefits of MedDiet on cardiovascular risk factors including obesity, hypertension, diabetes, and metabolic syndrome (4, 7–13). Therefore, it also decreases the risk for cardiovascular disease (CVD) morbidity and mortality (2, 8, 14–18).

Variants of the MedDiet have been consumed in more than 15 countries surrounding the Mediterranean Sea (19). The MedDiet includes high consumption of extra-virgin olive oil, fruits, vegetables, nuts, and legumes, unrefined whole grain and fish; a moderate intake of yogurt and fermented dairy, eggs, and poultry; moderate wine consumption with meals and low intake of processed and red meats and sweets (5, 14).

After a successful proof of concept, MedDiet has been shown to be applicable in non-Mediterranean countries as well, with supportive evidence for carrying on its cardioprotective role (20). From Australia to Japan, Chile and Iran as well as North-European countries, following the key principles of MedDiet has given great opportunities to various food types of local cuisines that can serve as great substitutes of food items that can be more easily found only in the Mediterranean region, thus enhancing its adherence on a global scale (20). Although a challenge, the applicability and transferability of the MedDiet in non-Mediterranean countries is desirable and supported by the current scientific evidence on its health benefits in non-Mediterranean countries. Some approaches such as the alternative MedDiet (16), and the recognition of this dietary pattern in the latest 2015 Dietary Guidelines for Americans are clear examples.

Emerging data from firefighters in the US, a population with significant health concerns, also support beneficial effects and potential for greater adoption of this eating pattern, despite living in a non-Mediterranean country. While less than 2% of firefighters currently follow the MedDiet, over 60% want to learn more about it and over 70% would be interested in an online nutritional platform (21). Controlled trials can quantify these benefits and determine the cost-effectiveness of implementation (22).

This paper will provide the rationale and need for Mediterranean Diet Nutrition Interventions (MDNI) in the US fire service. First, we will summarize data on CVD in firefighters and demonstrate that a major proportion of CVD morbidity and in particular, mortality is attributable to obesity and cardiometabolic risk clustering. We will then discuss nutritional challenges and opportunities in the fire service; the role of healthy diet in CVD prevention; in particular, the proven benefits of MedDiet; as well as the success of workplace interventions to change eating and lifestyle behavior. Moreover, we examine data revealing the role of obesity in increasing the risk of many cancers, while MedDiet reduces the risks of obesity, cancer, and other chronic diseases. Finally, we will use preliminary data from the fire service to support that an MDNI should improve key dietary habits, decrease weight gain, and improve firefighters’ CVD risk factor profile.

## CVD in the US Fire Service

Cardiovascular disease is the predominant cause of on-duty death and lifetime mortality in the US fire service. Sudden cardiac death (SCD) causes about 45% of on-duty fatalities. Strokes, aneurysms, and other CVD pathologies result in another 5% of on-duty deaths (23–26). There are an estimated 17–25 non-fatal, line-of-duty CVD events in the US fire service for every fatal on-duty CVD event (25). Thus, CVD is also a major cause of morbidity and disability (25, 27, 28). As in the general population, these CVD events are largely due to coronary heart disease (CHD) (23–25, 27). Finally, CHD accounts for 30% of all deaths and, thus, is the leading cause of lifetime mortality among US firefighters (29). Thus, interventions which prevent CVD are clearly the top priority for US fire service research. In this regard, the effect of MedDiet in CVD prevention is roughly equivalent to statin medications (e.g., Lipitor, Crestor) (30), while having added benefits on weight control, preventing diabetes, and decreasing the risk of cancer (31, 32).

Obesity and cardiometabolic risk clustering are well-established CVD risk factors in the general population, and as well obesity strongly promotes risk factor clustering as mediated by negative effects on blood pressure, metabolism, sleep-disordered breathing, and cardiac enlargement (28). In the fire service, obesity has documented adverse effects on: fitness, metabolic syndrome, left ventricular hypertrophy/cardiomegaly, incident CHD; on-duty CHD events, including SCD; injury risks/workers’ compensation costs; job-related disability and CVD retirements (24, 25, 27, 33–36).

The obesity problem in the US fire service has been increasing. Steadily within the worldwide obesity epidemic, about 40% of firefighters are now obese (25). A recent population-based investigation of both career and volunteer firefighters proved that the high obesity prevalence was due to excess adiposity rather than the misclassification of increased muscle mass (37). In fact, obesity was more prevalent when assessed by body fat measures compared to body mass index (BMI).

Even in a study of young fire recruits (mean age 26 years), 44% were overweight and 33% were obese. The obese subjects, compared to normal weight subjects, had an almost sevenfold greater risk of hypertensive blood pressure readings (38). Another recent investigation of young firefighters found 67% to be overweight or obese. Again, high BMI was associated with higher central blood pressure and increased arterial stiffness (39).

While the effects of excess weight on CVD are usually thought of as manifesting in middle-aged and older firefighters, we have recently linked obesity, hypertension, and other modifiable risks to fire service deaths in young firefighters. Among firefighters ≤45 years of age, at least two-thirds of on-duty SCD was related to preventable factors such as obesity and CHD (36). Moreover, we found surprising preliminary evidence of excess obesity among on-duty trauma deaths (burns, asphyxiation, and blunt trauma) compared with occupationally active control firefighters (36). These data suggest effective dietary intervention within an overall wellness strategy could reduce both CVD- and non-CVD-related morbidity and mortality.

## Existing Dietary Challenges and Opportunities

The high prevalence of obesity and CVD risk clustering in the fire service is multifactorial. First, shift work has been associated with weight gain, increased blood pressure, and worsening insulin resistance (25, 28). Moreover, shift work and unscheduled emergency calls, lead to irregular meal times, which increase the likelihood of choosing fast foods and other takeout foods. Thus, career firefighters are more likely to consume meals higher in sugars and saturated fats (40–42). These factors likely contribute to certain traditions around over-eating and less healthy choices common in fire service culture.

According to one of our recent studies, the two dietary factors that differed the most between obese and non-obese firefighters were obese firefighters’ higher consumption of sugary drinks and fast-food (41). These findings are consistent with those from the general population. Sweetened beverages are the largest contributors to added-sugar consumption in the US (43–45). In addition, investigations have associated increased fast-food consumption with obesity (46–48) and cardiometabolic risk (49).

Nonetheless, there are also good opportunities for intervention in the fire service and successes have been achieved with workplace approaches. Promoting Healthy Lifestyles: Alternative Models’ Effects was a prospective randomized controlled study of firefighter wellness, examining individual (one-on-one motivational counseling), and team health promotion approaches. Both interventions had beneficial effects on LDL cholesterol and exercise habits, compared with the control group (50). Moreover, after 1 year, both intervention groups demonstrated better weight control; more fruit/vegetable consumption and perceived greater well-being compared to controls (51). Even 4 years after the 12-month interventions, physical activity and nutrition remained improved compared with baseline (52).

There is also evidence that less intense wellness strategies have positive effects on fire service health. Poston et al. compared 10 fire departments that had implemented key Wellness Fitness Initiative (WFI) components (53) and compared them to 10 otherwise similar departments that had not implemented WFI approaches. Firefighters in the WFI departments had significantly lower BMI, body fat, waist circumference, and were over 40% less likely to be obese (53).

## MedDiet and CVD and Cancer Reduction

Numerous studies have demonstrated the effectiveness of MedDiet in reducing all-cause mortality (54–56), CVD morbidity and mortality, and cancer mortality (1–6, 57). These benefits likely derive from effects on intermediate states such as inflammation hypertension, obesity, metabolic syndrome, and diabetes mellitus (4, 7–10). Given these clear benefits, the latest US government nutritional guidelines recognize and recommend the MedDiet as a healthy option for Americans (58). Most nutrition experts recognize that there are many healthy eating patterns found across the globe, including Asian and vegetarian options, but there is a consensus that the “Mediterranean diet reigns supreme” considering all the evidence and pros and cons of each (59).

Consistent evidence of benefits from MedDiet has inspired MDNI. In the classic Lyon Heart study, subjects with a history of myocardial infarction were randomized to an MDNI or control diet (59). The MDNI mimicked a traditional Cretan diet with less red meat, but more fruit, vegetables, fish, and margarine. The randomized controlled trial (RCT) was stopped early because of excessive morbidity/mortality in the control arm, while the MDNI lowered the risk of recurrent heart disease by 50–70% during follow-up (60).

Mediterranean Diet Nutrition Intervention trials for primary prevention have also provided very promising results. Metabolic syndrome, a precursor of heart disease, affects over 25% of career firefighters (61). A 2-year randomized trial of MDNI reversed metabolic syndrome in 2/3 of the intervention group, whereas metabolic syndrome persisted in more than 80% of the controls (62). The most notable MDNI trial to date is PREDIMED in Spain (17). Beginning in 2003, PREDIMED ultimately randomized over 7,000 participants at high CVD risk, but without known CVD, to one of three interventions: an MDNI supplemented with extra-virgin olive oil; an MDNI supplemented with mixed nuts or a low-fat diet. The most recent data from PREDIMED provide the highest quality scientific evidence thus far of MedDiet benefits. After an average of 4.8 years of follow-up, subjects given the MDNI with extra-virgin olive oil had a 40% reduction in the incidence rate of new-onset diabetes (63), and a 30% decrease in major CVD events compared to control subjects prescribed a low-fat diet (14). In addition, PREDIMED has already documented other benefits, including decreased risk of breast cancer (64) and cognitive decline (65).

It is important to note that MedDiet is associated with significantly decreased cancer risks (6, 66, 67). First, although there are diverse workplace exposure and other causes of cancer, obesity is a major and modifiable risk factor for many types of cancer (34). Second, obesity is highly prevalent among US firefighters (25, 37) and third, MedDiet can reduce body weight and help maintain lower body weights. Finally, following a MedDiet produces large and significant decreases in cancer risks (1, 68–70). According to the most recent study, cancer is the second leading cause of lifetime mortality among US firefighters and accounts for over 25% of deaths (29). Therefore, long-term Mediterranean dietary changes in the fire service should positively impact firefighters’ cancer burden.

## MedDiet and Workplace Behavioral Change

As we recently summarized in published reviews of MedDiet and the workplace, experience with worksite MDNIs is limited, but the evidence is quite positive (66, 67). Shai et al. have completed the only RCT at an Israeli nuclear facility, where over 300 obese participants were randomly assigned to: a low-fat, restricted-calorie diet; a Mediterranean, restricted-calorie diet; or a low-carbohydrate diet without calorie restriction (71). The 2-year intervention included a spousal education program and changes in the workplace cafeteria (72). After 6 years, the total weight loss was greatest and most significant for the Mediterranean group (3.1 kg), whereas the other two groups gained back most of their weight (73). Moreover, the Mediterranean group had the greatest persistent reductions in triglyceride and cholesterol levels from baseline.

In a Chilean factory, a 1-year, uncontrolled MedDiet intervention using education and changing the employer’s cafeteria significantly improved MedDiet scores, waist circumference, HDL, blood pressure, and metabolic syndrome prevalence among the participants (2).

Carey et al. performed an uncontrolled pilot study of a 12-week low-glycemic nutritional program including some MedDiet principles in 10 men from one platoon of the Buffalo, NY Fire Department (74). At baseline, 70% had metabolic syndrome and an average of 3.2 metabolic syndrome risk factors. Upon completion of the pilot, the prevalence of metabolic syndrome and risk factors had decreased significantly: 30% and 1.9 metabolic syndrome risk factors, respectively.

## Preliminary Data

### Modified MedDiet Score and Cardiovascular Risk in Midwestern Firefighters

We previously investigated the dietary habits of 780 Midwestern firefighters using a modified Mediterranean diet score (mMDS) derived from a comprehensive lifestyle questionnaire. Greater adherence to MedDiet as measured by the mMDS was significantly associated with improvements in body fat, metabolic syndrome, LDL- and HDL-cholesterol, weight gain, and aerobic fitness (41). Firefighters with greatest adherence to MedDiet showed a 35% decreased risk of metabolic syndrome and 43% lower risk of weight gain compared to the bottom quartile of mMDS. Associations for improved HDL, total cholesterol/HDL, and body fat remained significant even after adjustments for age, BMI, and physical activity levels (36).

### National Surveys of International Association of Fire Fighters (IAFF) Members

Dietary change is more likely to happen if the change strategy is acceptable to the target population and addresses perceived knowledge gaps. Therefore, in collaboration with the IAFF (21), we have conducted national surveys of firefighters. We found that 71% of IAFF members do not currently follow any particular dietary plan, and less than 2% report that they currently follow the MedDiet. However, over 70% want to learn more about the MedDiet; and they most frequently rated the MedDiet description as their favorite and gave it better rankings (*p* < 0.001) compared to the Paleo, Atkins, and other popular diets. Diverse and colorful flavors; the ability to enjoy lean meats in moderation; healthy fats and proteins from olive oil, nuts, fish, and the temperate wine consumption in moderation make the MedDiet an attractive and enjoyable option. Most important, the MedDiet does not require completely giving up any specific food and is, therefore, accessible and acceptable for adoption and long-term adherence among diverse groups, including the fire service. The weight status and the opinions expressed by career firefighters results strongly support the need for and a positive reception to potential MDNIs.

## Discussion

We know that more than one of every three firefighters in the US is obese and SCD is the number one line of duty killer-far ahead of burns and dangerous gases in fire smoke. We are also aware that fire department environments may promote and reinforce poor eating habits and thus, may inadequately increase CVD and obesity risks. Obviously, given the positive effects of MedDiet on CVD, obesity control, and cancer, it is more important than ever to more widely disseminate and implement MedDiet in the US fire service to improve firefighter health and longevity. We step–step provided the evidence necessary to promote behavioral change strategies in the fire service and modify the existing food culture, with the ultimate purpose to be getting more firefighters and their families to adopt the principles of the MedDiet to decrease their risks of chronic disease.

All our research stated above has provided the evidence needed and put the rationale and the basis; and with an unprecedented success, the US Department of Homeland Security awarded our team in 2015 with a $ 1.5-million, 3-year, competitive research grant entitled “Feeding America’s Bravest: MedDiet-Based Interventions to Change Firefighters’ Eating Habits and Improve Cardiovascular Risk Profiles” to conduct Mediterranean Diet Nutritional Interventions in selected US firefighters. Namely, our trials consist of two parallel designs. First, regarding the career firefighters, we have developed multi-pronged, MDNI behavior change strategies including: diet/lifestyle education; discounted access to key MedDiet foods; electronic education platforms and reminders. MDNI components have been refined *via* surveys, literature review, and local/national firefighter input including labor/management and fire service focus groups, and as a result of the above refinements, our team has developed original firefighter-specific MedDiet pyramid, food shopping guides and recipes many of which were designed and created based on firehouse favorites. Second, we have developed an online, open trial as a demonstration project to help change volunteer firefighters’ health habits and improve cardiovascular risk profiles using a MedDiet intervention strategy. The online tools include firefighter-centered guidelines for eating and exercise, firefighter-favorite recipes, food shopping tips, guides for eating out and on the go, running healthy meetings and a series of short instructional videos, among other resources.

Nutrition and medical experts agree that following a Mediterranean-style diet improves health. However, the health system has had very limited effectiveness in changing Americans’ eating and other lifestyle behaviors, while limited evidence suggests that workplace-based nutrition interventions can be beneficial. Building on our prior work with firefighters and the above rationale, our funded projects seek to establish the effectiveness of behavioral change strategies in the fire service to modify the existing food culture. Our goal is to motivate firefighters and their families to adopt key features of the MedDiet at work and home through education, participation, and incentives.

Overall, our team aims to create comprehensive, accessible, and sustainable programs around the time-honored and scientifically proven principles of the Traditional MedDiet that after a successful proof of concept, can be disseminated to nationally to the rest of the US fire service, as well as other workplaces and schools in the future. Already, we are working with two other outstanding employers who are preparing to adopt and incorporate healthy dietary workplace initiatives: overall, our team aims to create a comprehensible, approachable, and sustainable program around the time-honored and scientifically proven principles of the Traditional MedDiet that after a successful proof of concept, can be disseminated to the rest of the fire service and other workplaces and schools in the future: law enforcement officers at the Broward County Sheriff’s Office (Florida) and the automobile manufacturer, SEAT, in Spain (75).

## Conclusion

The ultimate purpose of this work is to get more firefighters and their families to adopt the principles of the MedDiet to decrease their risks of chronic disease.

## Author Contributions

All authors contributed equally to designing and drafting the manuscript.

## Conflict of Interest Statement

SK reports grants from US Dept. of Homeland Security, non-financial support from Barilla America, non-financial support from California Almond Board, non-financial support from Arianna Trading Company, non-financial support from Innoliva/Molina de Zafra, during the conduct of the study; personal fees from Medicolegal Consulting, personal fees from Mediterranean Diet Roundtable, outside the submitted work (22). MK and MS-P declare no competing interests.
